# Bottom-up-then-up-down Route for Multi-level Construction of Hierarchical Bi_2_S_3_ Superstructures with Magnetism Alteration

**DOI:** 10.1038/srep10599

**Published:** 2015-06-01

**Authors:** Chengzhen Wei, Lanfang Wang, Liyun Dang, Qun Chen, Qingyi Lu, Feng Gao

**Affiliations:** 1 State Key Laboratory of Coordination Chemistry, School of Chemistry and Chemical Engineering; Collaborative Innovation Center of Advanced Microstructures, Nanjing National Laboratory of Microstructures, Nanjing University, Nanjing 210093, P. R. China; 2 Department of Materials Science and Engineering, Nanjing University, Nanjing 210093, P. R. China

## Abstract

A bottom-up-then-up-down route was proposed to construct multi-level Bi_2_S_3_ hierarchical architectures assembled by two-dimensional (2D) Bi_2_S_3_ sheet-like networks. BiOCOOH hollow spheres and flower-like structures, which are both assembled by 2D BiOCOOH nanosheets, were prepared first by a “bottom-up” route through a “quasi-emulsion” mechanism. Then the BiOCOOH hierarchical structures were transferred to hierarchical Bi_2_S_3_ architectures through an “up-down” route by an ion exchange method. The obtained Bi_2_S_3_ nanostructures remain hollow-spherical and flower-like structures of the precursors but the constructing blocks are changed to 2D sheet-like networks interweaving by Bi_2_S_3_ nanowires. The close matching of crystal lattices between Bi_2_S_3_ and BiOCOOH was believed to be the key reason for the topotactic transformation from BiOCOOH nanosheets to 2D Bi_2_S_3_ sheet-like nanowire networks. Magnetism studies reveal that unlike diamagnetism of comparative Bi_2_S_3_ nanostructures, the obtained multi-level Bi_2_S_3_ structures display S-type hysteresis and ferromagnetism at low field which might result from ordered structure of 2D networks.

One-dimensional (1D) nanostructures such as tubes, rods and wires have aroused intensively attention due to their distinct properties and potential applications^1^,^2^,^3^. In comparison with the single 1D nanostructures, complex functional architectural structures constructed by 1D building blocks supply possible opportunities to study their unique properties due to their complex structures and thus have received broad attention in the materials fields. Up to now, some architecture assembled by 1D nanostructures were successfully prepared^4^,^5^,^6^. However, the synthesis of novel complex architectural structures via a simple, mild and effective way still remains a great challenge. Topotatic transformation was developed on the basis of topological chemical method for the synthesis of crystals with special morphologies and applications^7^. Through reaction designs, crystals of certain shapes are obtained first and then under specific conditions the crystals would transform to other crystals without changing the spatial morphology but the crystal structures^8^,^9^,^10^,^11^. This topological transformation process can not only increase the predictability of the produced materials’ space structures but also make assembly more diversified to construct special structures.

Bismuth sulfide (Bi_2_S_3_), as an important layer-structured semiconductor with a direct band gap of 1.3 eV, has potential applications in many fields including catalysis, sensor, optoelectronic nanodevice and lithium ion battery^12^,^13^. Recently, many efforts have been devoted to the controlled preparation of complex architectural Bi_2_S_3_ structures and different Bi_2_S_3_ architectures such as snowflake-like, core-shell microspheres, sheaf-like and crossed nanofabrics have been prepared^14^,^15^,^16^,^17^. However, Bi_2_S_3_ with multi-level architectures based on 1D nanostructures have not been accomplished yet. Herein, we present a topotactic transformation route for multi-level Bi_2_S_3_ architectures assembled by 2D sheet-like networks interweaved by 1D nanowires. Hollow spherical and flower-like BiOCOOH structures assembled by BiOCOOH nanosheets were first prepared by a “bottom-up” route through a “quasi-emulsion” mechanism. Then the BiOCOOH hierarchical structures were transferred to multi-level hierarchical Bi_2_S_3_ architectures through an “up-down” route by an ion exchange method. The obtained Bi_2_S_3_ nanostructures remained hollow-spherical and flower-like structures of the precursors but the constructing blocks changed to 2D sheet-like networks interweaved by Bi_2_S_3_ nanowires. Magnetism studies reveal that the novel multi-level structures display different magnetic behaviors from the diamagnetism of the comparative Bi_2_S_3_ structures.

## Results

**Figure 1:**
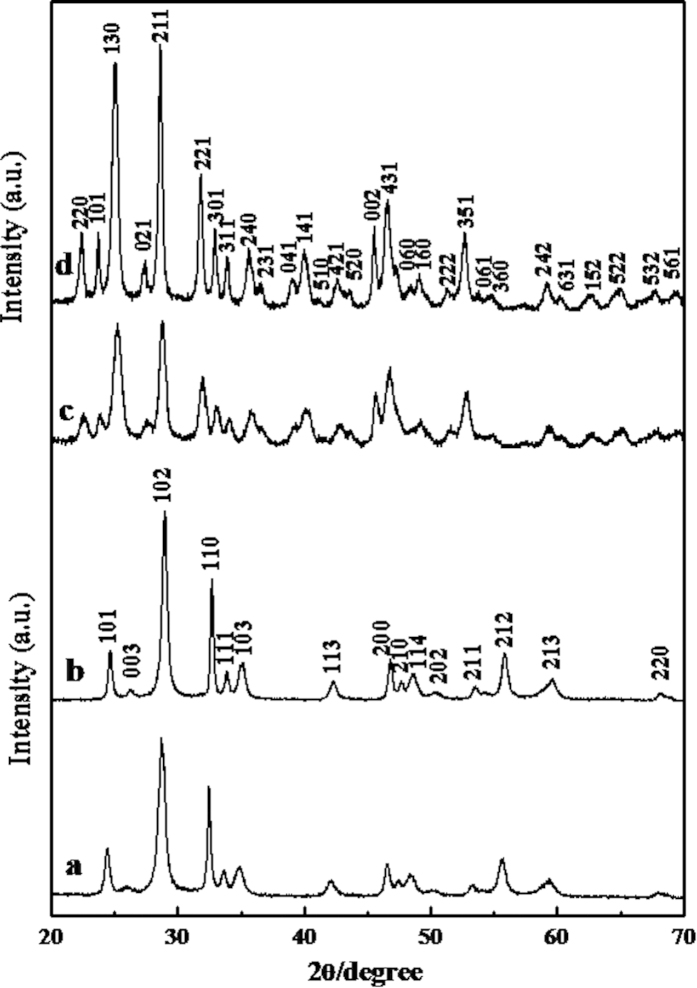
XRD patterns of the obtained products: (**a**) hierarchical BiOCOOH hollow spheres prepared with the addition of glycerol; (**b**) flower-like BiOCOOH structures without the addition of glycerol; (**c**) Bi_2_S_3_ multi-level superstructures transformed from hierarchical BiOCOOH hollow spheres and (**d**) Bi_2_S_3_ multi-level superstructures transformed from BiOCOOH flower-like structures.

**Figure 2:**
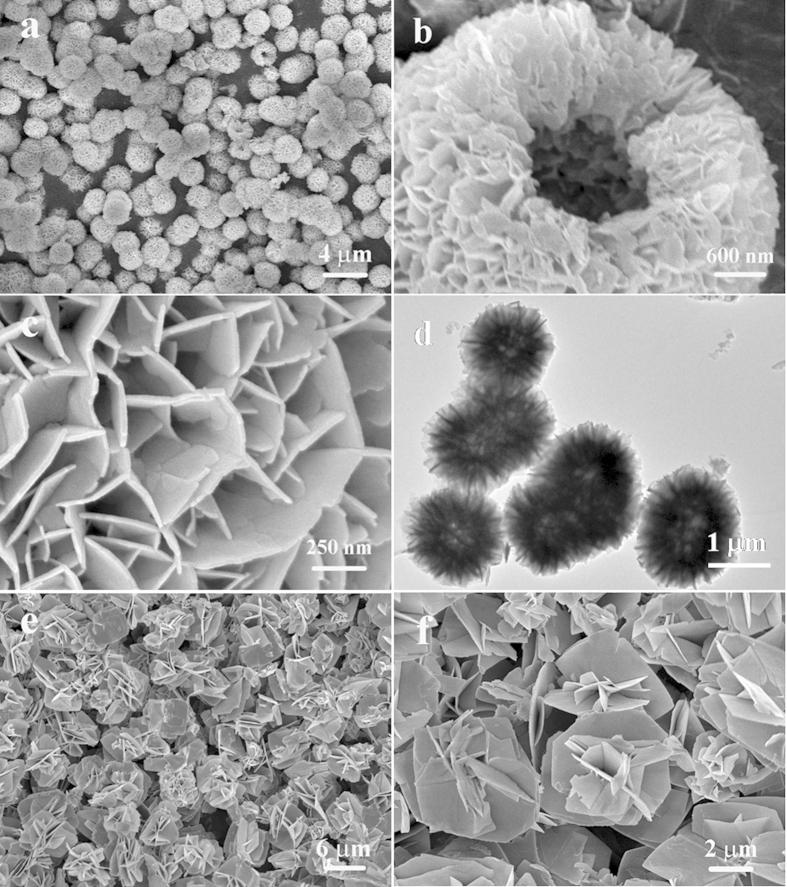
(a, b) SEM and (c, d) TEM images of hierarchical BiOCOOH hollow spheres prepared with the addition of glycerol; (e, f) SEM images of flower-like BiOCOOH structures without the addition of glycerol.

The BiOCOOH structure were prepared by solvothermally treating Bi(NO_3_)_3_ in glycerol, H_2_O and DMF mixture. Figure 1a displays XRD pattern of the as-prepared product, in which all the diffraction peaks can be indexed to tetragonal BiOCOOH (JCPDS card No. 35-0939, a =  b = 3.90 Å and c = 10.2 Å). No diffraction peaks corresponding to other impurities are detected, indicating the high purity of the product. The morphology of the sample was observed by scanning electron microscopy (SEM) and transmission electron microscopy (TEM). As an SEM image in Fig. 2a shows, the product consists of uniform BiOCOOH spheres with an average diameter of 2 μm. These BiOCOOH spheres are hierarchical hollow structures assembled by interlaced nanosheets. Figure 2b clearly shows the hollow interior and Fig. 2c demonstrates that the shell of the hollow sphere is assembled by nanosheets with thickness of several nanometers. A TEM image shown in Fig. 2d, in which a contrast between the boundary and the center of the spheres can be clearly seen, provides the further evidence for the hollow nature of the hierarchical structure. Glycerol would play an important role for the formation of hollow spheres. Without the addition of glycerol, no hierarchical BiOCOOH hollow spheres but flower-like BiOCOOH structures assembled by sheet-like BiOCOOH (XRD pattern is shown in Fig. 1b) were obtained as SEM images in Fig. 2e and f display. EDX were also conducted to demonstrate the purity of the products. From the EDX patterns shown in Figures S1a and b, only C, O, and Bi along with Au can be detected, in which Au signals are attributed to gold spraying for SEM investigation, confirming the pure nature of the obtained hierarchical structures.

**Figure 3:**
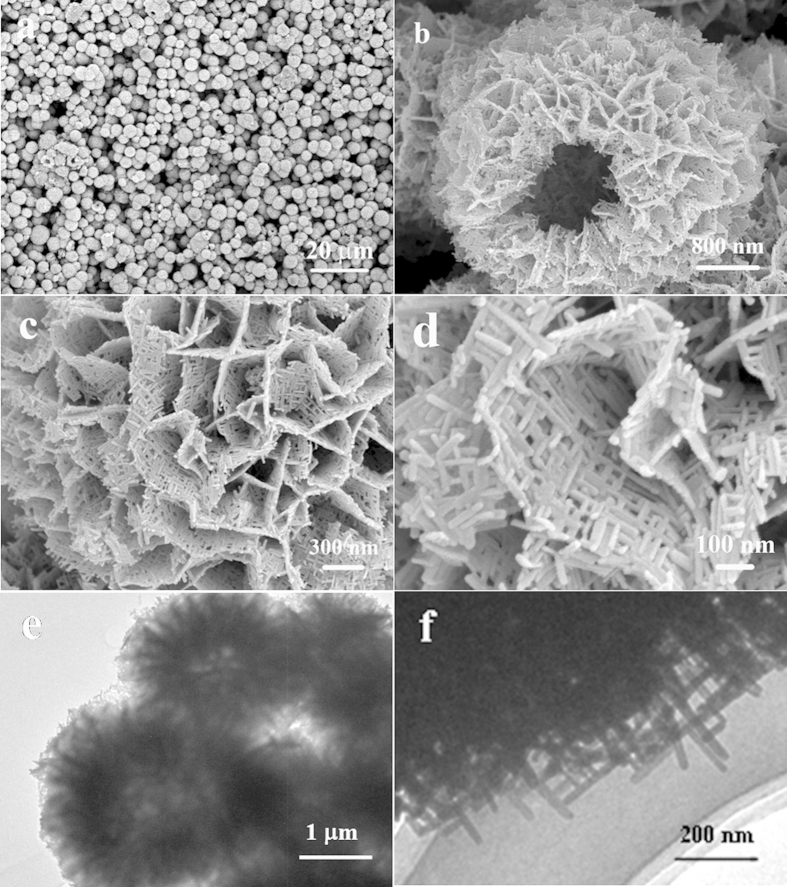
(a~d) SEM images with different magnifications and (e, f) TEM images of the Bi_2_S_3_ multi-level superstructures transformed from hierarchical BiOCOOH hollow spheres.

The acquirement of hierarchical BiOCOOH structures brings us opportunities to construct multi-level architectures through topotactic transformation. In this study, the hierarchical BiOCOOH structures were transformed to Bi_2_S_3_ complex architectures through an “up-down” ion exchange method by mixing thioacetamide (TAA) and BiOCOOH in 20 mL of H_2_O, followed by hydrothermal treatment at 120 °C for 12 h. XRD pattern of the product is shown in Fig. 1c and the diffraction peaks can be indexed to pure orthorhombic Bi_2_S_3_ (JCPDS 17-0320: a = 11.14 Å, b = 11.30 Å, and c = 3.98 Å), indicating that the BiOCOOH structures were completely transformed to Bi_2_S_3_ crystals. Figure 3 displays SEM and TEM images of the obtained Bi_2_S_3_ structures. A panoramic SEM image presented in Fig. 3a reveals that the Bi_2_S_3_ sample is composed of microspheres in large scale with an average diameter of about 2 μm. The hollow nature of the Bi_2_S_3_ spheres can be clearly seen from Fig. 3b. Unlike the parent BiOCOOH hollow spheres which are assembled by smooth BiOCOOH nanosheets, the Bi_2_S_3_ hollow microspheres are constructed by 2D sheet-like networks as magnified SEM images show in Fig. 3c and d, which demonstrates that the Bi_2_S_3_ hollow spheres are multi-leveled superstructures. The 2D sheet-like Bi_2_S_3_ networks are interwoven by crossed Bi_2_S_3_ nanorods with long range order. The nanorods are uniform with a diameter of about 20 nm and the angle between the adjacent nanorods is about 90°. It is worth to mention that such multi-leveled complex hollow Bi_2_S_3_ superstructures assembled by 2D disc-like networks have never been reported before. The corresponding TEM images of the microspheres presented in Fig. 3e and f further confirm that the Bi_2_S_3_ structures are hollow by clearly displaying a contrast between the dark edges and the pale center and made of crossed nanorods with a diameter of about 20 nm. EDX and XPS were both used to demonstrate the formation of the products. From the EDX pattern shown in Figures S1c, only Bi and S along with Au can be detected, confirming the pure nature of the obtained hierarchical structures. XPS characterizations as shown in Fig. S2 also suggest the formation of Bi_2_S_3_.

**Figure 4:**
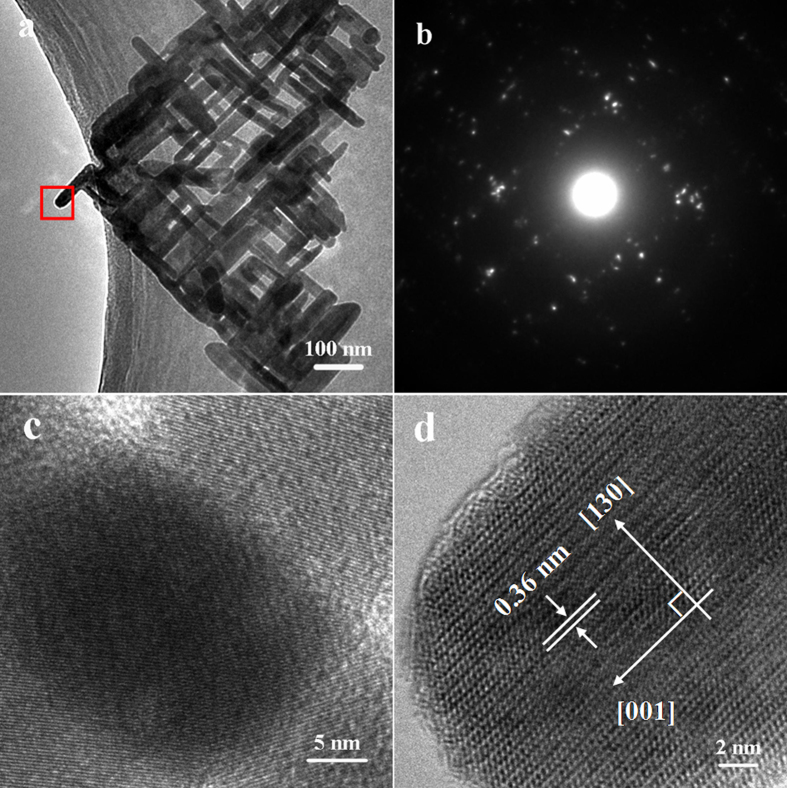
(a, b) TEM image and the related SAED pattern of a 2D sheet-like Bi_2_S_3_ nanorod network by sonicating the complex Bi_2_S_3_ hollow microspheres; (**c**) An HRTEM image of the crossed junction of Bi_2_S_3_ nanorod network; (**d**) An HRTEM image of a single Bi_2_S_3_ nanorod.

The crystalline structure of the 2D Bi_2_S_3_ sheet-like networks obtained by sonicating the complex Bi_2_S_3_ hollow microspheres was further characterized by TEM and HRTEM. As shown in Fig. 4a, 2D sheet-like networks can be observed, which confirms that the 2D sheet-like Bi_2_S_3_ networks are interwoven by crossed Bi_2_S_3_ nanorods with a diameter of about 20 nm and the angle between the adjacent nanorods of about 90°. The SAED pattern corresponding to the whole 2D sheet-like networks shown in Fig. 4a was presented in Fig. 4b, which displays orderly arranged spots with a tetragonal-like symmetry, reflecting that the Bi_2_S_3_ nanorods are interwoven into the network in a tetragonal symmetry. It also reveals that the perpendicularly aligned nanorods might have preferential growth direction of [001]. Figure 4c and d display HRTEM images of a crossed nanorod junction and an individual nanorod (the area marked by the red rectangle in Fig. 4a), respectively, both exhibiting clear lattice fringes and suggesting the high crystallinity of the 2D networks. In Fig. 4d, the typical HRTEM image of the individual Bi_2_S_3_ nanorod display the lattice spacing of about 0.36 nm, which is consistent with the spacing of (130) planes of orthorhombic Bi_2_S_3_. Although the fringes of the (001) facet are not found in the image, it can still be proposed that the nanorods grow along the [001] direction, which is perpendicular to (130) facets.

## Discussions

**Figure 5:**
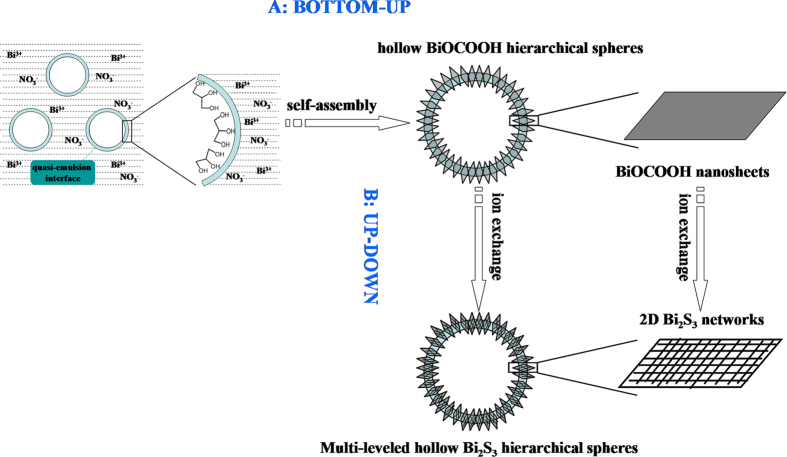
Scheme of the “bottom-up-then-up-down” route for the construction of hierarchical Bi_2_S_3_ hollow spheres assembled by 2D Bi_2_S_3_ networks.

In the reaction system glycerol plays an important role for the formation of hollow BiOCOOH spheres. Without the addition of glycerol, when Bi(NO_3_)_3_ was solvothermally treated in H_2_O and DMF, no BiOCOOH hollow spheres but flower-like BiOCOOH structures were obtained. Based on the experiment results, a “quasi-emulsion-templated” mechanism is proposed for the BiOCOOH hollow spheres in glycerol and water mixture^18^,^19^,^20^. It is known that although alcohols and water are infinitely miscible, the alcohol aqueous solutions are inhomogeneous due to the fact that the alcohols tend to self-assemble in the aqueous solution^21^. In the present case, when glycerol was mixed with water, we speculated that due to the self-assembly of glycerol molecules, a uniform quasi-microemulsion might form. Polyols (e.g. EG, glycerol) are well known to have strong coordination ability towards metal ions^22^,^23^,^24^. So, when Bi(NO_3_)**·**5H_2_O was added into the mixture of water and glycerol, the coordination between Bi^III^ and glycerol would make Bi^III^ gather onto the surface of the emulsified spheres. As reaction temperature increases, DMF hydrolyze into formic acid to react with Bi^III^ to form BiOCOOH. Driven by the minimization of interfacial energy and templated by the emulsified spheres, the sheet-like BiOCOOH aggregate around the emulsified spheres and lead to the formation of hierarchical BiOCOOH hollow spheres. The formation process of BiOCOOH hollow spheres can be illustrated as shown in Fig. 5A.

**Figure 6:**
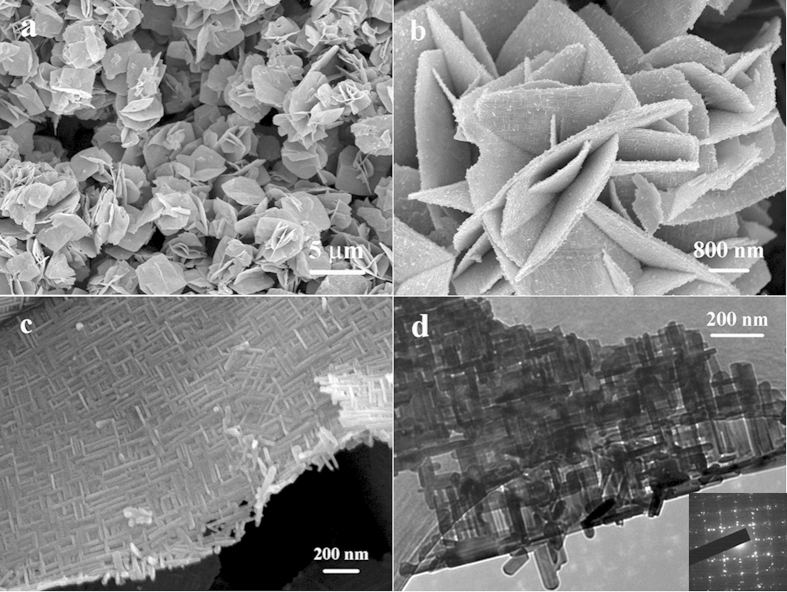
(a–c) SEM images with different magnifications and (**d**) TEM image and the related SAED pattern of the Bi_2_S_3_ multi-level superstructures transformed from BiOCOOH flower-like structures.

The crystal lattice matching would be the key factor for the topotactic transformation from BiOCOOH to the multi-level Bi_2_S_3_ architectural structures. It is commonly accepted that Bi_2_S_3_ crystals can easily grow along its *c*-axis into 1D nanostructures^25^. In the present preparation of Bi_2_S_3_ architectural structures, the crystal lattice relationship between the *a*- or *b*- axis of tetragonal BiOCOOH (a = b = 3.9145 Å) and the *c*-axis of orthorhombic Bi_2_S_3_ (c = 3.981 Å) could be responsible for the formation of 2D Bi_2_S_3_ sheet-like networks^9^,^26^. When sulfur ions were released from TAA, they may replace O^2−^ and HCOO^-^ ions in BiOCOOH nanosheets to form [001]-oriented Bi_2_S_3_ nanorods lying on the top surfaces of BiOCOOH nanosheets. The [001]-oriented Bi_2_S_3_ nanorods would have a tendency to oriented along the two perpendicular [100] and [010] directions of BiOCOOH because of the close lattice matching between the *a*- or *b*- axis of BiOCOOH and the *c*-axis of Bi_2_S_3_, which finally leads to the formation of 2D Bi_2_S_3_ sheet-like networks. The topotactic transformation process remains the spatial structure of the precursor and leads to the multi-level construction of complex Bi_2_S_3_ hollow microspheres assembled by 2D sheet-like networks. The topotactic transformation process can be confirmed by reducing the reaction times. During the transformation from BiOCOOH to Bi_2_S_3_, intermediate mixtures BiOCOOH and Bi_2_S_3_ were detected when the reaction time was 1 h and 3 h. Corresponding XRD patterns and SEM images were presented in Fig. S3 and S4. From the XRD patterns, it can be seen that with the increase of the reaction time, the peak intensities of Bi_2_S_3_ became stronger, indicating that more Bi_2_S_3_ crystals formed. From the SEM investigations, the form Bi_2_S_3_ formed on the surface of BiOCOOH nanosheets in an oriented direction. The process can be further proved by transforming BiOCOOH flower-like structure to Bi_2_S_3_ superstructures. As SEM and TEM images shown in Fig. 6, after being treated with thioacetamide (TAA) under hydrothermal conditions at 120 °C, the flower-like BiOCOOH structures were also transformed to multi-level Bi_2_S_3_ flower-like superstructures assembled by 2D sheet-like networks. In the other hand, when we tried to transform BiOCOOH structures to form Bi_2_Te_3_ structures, such novel multi-level structures can’t be obtained. Fig. S5 displays the XRD patterns of the products prepared by treating BiOCOOH structures with tellurium sources under hydrothermal conditions, which confirms that Bi_2_Te_3_ crystals have been obtained (JCPDS card No. 08-027, a = b = 4.43 Å and c = 29.91 Å). However, the SEM investigations (Fig. S6) reveal that although the obtained Bi_2_Te_3_ structures remain the spatial structures of the parent BiOCOOH structures but the building blocks are Bi_2_Te_3_ nanosheets with smooth surfaces and don’t show 2D orderly network structure as Bi_2_S_3_. These results indirectly confirm that the crystal lattice matching is responsible for the multi-level superstructures.

**Figure 7:**
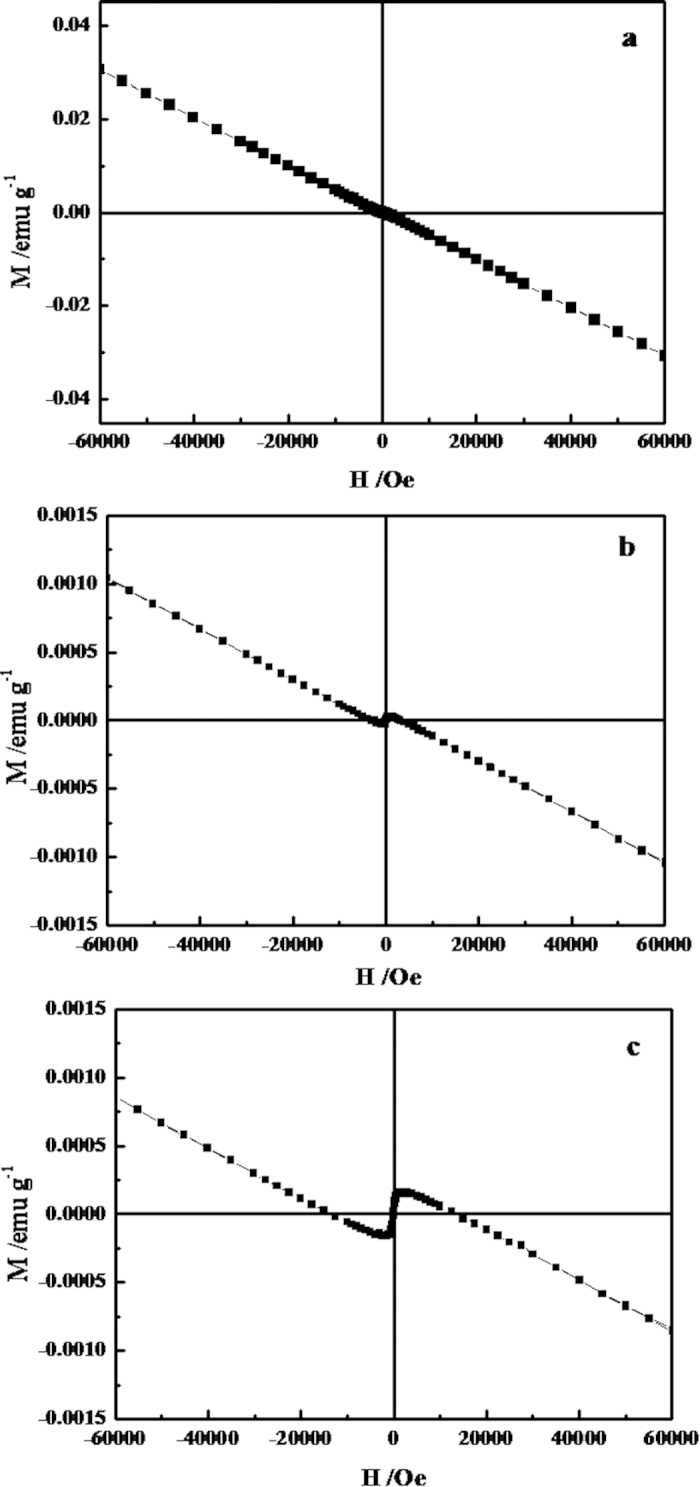
M-H curves of (a) the comparative Bi_2_S_3_ sample; (**b**) the multi-level hollow Bi_2_S_3_ spheres and (**c**) the multi-level follower-like Bi_2_S_3_ structures.

Magnetic properties of the multi-level Bi_2_S_3_ hollow spheres and flower-like structures assembled by 2D networks were investigated in this study. For comparisons, Bi_2_S_3_ nanostructures assembles by nanorods were also prepared under hydrothermal conditions by treating the aqueous solution of Bi(NO_3_)_3_ and thiourea at 160 °C for 16 h, whose SEM images are shown in Fig. S7. Figure 7 shows the magnetic hysteresis (*M-H*) loops at room temperature of all the three samples including the comparative Bi_2_S_3_ nanostructures, the obtained multi-level Bi_2_S_3_ hollow spheres and flower-like structures. For the comparative Bi_2_S_3_, a diamagnetic *M-H* curve was observed, revealing that the conventional Bi_2_S_3_ material is intrinsic diamagnetic. Interestingly, for the novel multi-level Bi_2_S_3_ superstructures, the *M-H* curve transits to be S-type hysteresis, suggesting the appearance of ferromagnetic long-range ordering in the multi-level Bi_2_S_3_ superstructures at low fields. Fig. S8 displays enlarged *M-H* curve from -2000 Oe ~2000 Oe, exhibiting typical ferromagnetic hysteresis loops at room temperature with coercivity forces of about 84 Oe and 40 Oe for superstructured Bi_2_S_3_ hollow spheres and flowers, respectively. At larger magnetic field part, the obtained Bi_2_S_3_ superstructures also exhibit diamagnetic behaviors arising from the intrinsic Bi_2_S_3_ because of the decrease of magnetization. In the experiments, all the used chemicals were analytic grade and all the three samples were prepared through the same route and the same treatment procedure. So, although to reach a clear conclusion requires further investigations, the difference in the magnetic properties of the comparative Bi_2_S_3,_ the multi-level Bi_2_S_3_ hollow spheres and flower-like structures would probably rise from their different structures.

In summary, novel multi-level Bi_2_S_3_ superstructures, hollow spheres and flower-like structures constructed by 2D sheet-like networks, have been successfully synthesized using BiOCOOH as the precursors through a “bottom-up-then-up-down” topotactic transformation process. The glycerol in the reaction system plays an important role for the formation of hierarchical BiOCOOH hollow structures and the crystal lattice matching is demonstrated to be responsible for the topotactic transformation from BiOCOOH to Bi_2_S_3_ to construct the multi-level hierarchical structures. Unlike diamagnetism of comparative Bi_2_S_3_ nanostructures, the obtained multi-level Bi_2_S_3_ structures display ferromagnetism at low field.

## Methods

### Synthesis of hierarchical BiOCOOH structures

All chemical agents were of analytical grade and purchased from Shanghai Chemical Reagent Factory and used directly without further purification. For the synthesis of BiOCOOH hollow spheres, 1.0 mmol of Bi(NO_3_)**·**5H_2_O was dissolved in 12 mL of glycerol, 3 mL of H_2_O and 5 mL of DMF to form a stable transparent solution under vigorously magnetic stirring. Then the obtained homogeneous solution was transferred into a 50 mL Teflon-lined stainless steel autoclave and sealed to heat at 160 °C for 12 h. After heated, the autoclave was cooled to room temperature naturally. The obtained precipitate was collected and washed with ethanol and de-ionized water in sequence by centrifugation, and then dried at 50 °C for 12 h. For the synthesis of flower-like BiOCOOH structures, 1.0 mmol of Bi(NO_3_)**·**5H_2_O was added to 15 mL of H_2_O and 5 mL of DMF under magnetic stirring for several minutes. Other experimental conditions remained the same as that of BiOCOOH hollow spheres.

### Synthesis of multi-level Bi_2_S_3_ architectures

1 mmol of BiOCOOH (hierarchical hollow spheres or flower-like structures) and 1.5 mmol of thioacetamide (TAA) was added into 20 mL of H_2_O under magnetic stirring for several minutes. The solution was transferred into a 50 mL Teflon-lined stainless steel autoclave and sealed to heat at 120 °C for 12 h, then cooled to room temperature naturally. The obtained precipitate was collected and washed with ethanol and de-ionized water in sequence by centrifugation, and then dried at 50 °C for 12 h.

### Characterizations

The phases of the obtained samples were characterized by X-ray diffraction (XRD) on a Shimadzu XRD-6000 powder X-ray diffractometer with Cu K_α_ radiation (λ = 1.5418 Å). The morphologies of the samples were investigated on a Hitachi S-4800 field-emission scanning electron microscope (FE-SEM) at an acceleration voltage of 10.0 KV. Transmission electron microscopy (TEM), high-resolution TEM (HRTEM) images and the corresponding selected area electron diffraction (SAED) were obtained on the JEOL JEM-2100 transmission electron microscope at an acceleration voltage of 200 KV. The magnetic measurements were carried out using a SQUID magnetometer (Quantum Design) at 300 K.

## Additional Information

**How to cite this article**: Wei, C. *et al*. Bottom-up-then-up-down Route for Multi-level Construction of Hierarchical Bi_2_S_3_ Superstructures with Magnetism Alteration. *Sci. Rep.* **5**, 10599; doi: 10.1038/srep10599 (2015).

## Supplementary Material

Supplementary Information
