# Supplementary material for: Bottom-up-then-up-down Route for Multi-level Construction of Hierarchical Bi2S3 Superstructures with Magnetism Alteration
Source: Sci Rep. 2015 Jun 1;5:10599. doi: 10.1038/srep10599 (PMC4450597; doi:10.1038/srep10599)
Supplement: Supplementary Information [file srep10599-s1.doc]

**Supporting Information (SI)**

**Bottom-up-then-up-down Route for Multi-level Construction of Hierarchical Bi_2_S_3_ Superstructures with Magnetism Alteration****

Chengzhen Wei, Lanfang Wang, Liyun Dang, Qun Chen, Qingyi Lu, Feng Gao

Figure S1 EDX spectra of the obtained BiOCOOH and Bi_2_S_3_ nanostructures: (a) BiOCOOH Hollow micro-spheres; (b) BiOCOOH Flower-like structures; (c) Bi_2_S_3_ Hollow micro-spheres; and (d) Bi_2_S_3_ Flower-like structures.

Figure S2 XPS spectra of the obtained Bi_2_S_3_ nanostructures: (a) Hollow micro-spheres; and (b) Flower-like structures.

Figure S3 XRD patterns of the products prepared by treating BiOCOOH and TAA under hydrothermal conditions for different times: (a) 1 h; (b) 3 h.


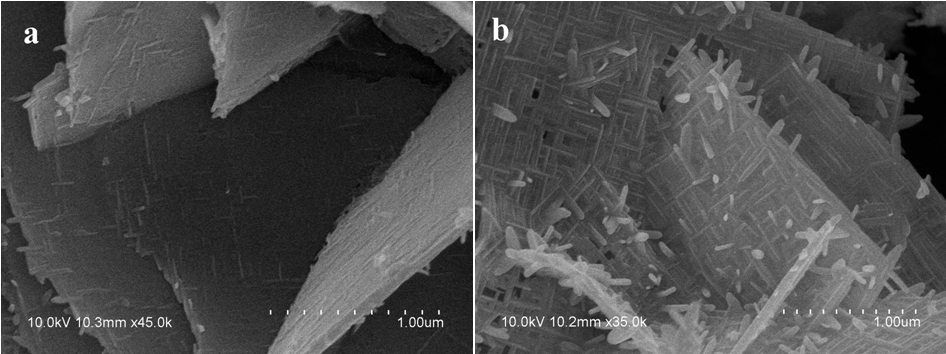


Figure S3 SEM images of the products prepared by treating BiOCOOH and TAA under hydrothermal conditions for different times: (a) 1 h; (b) 3 h.

Figure S5 XRD patterns of the products prepared by treating BiOCOOH structures with tellurium sources under hydrothermal conditions


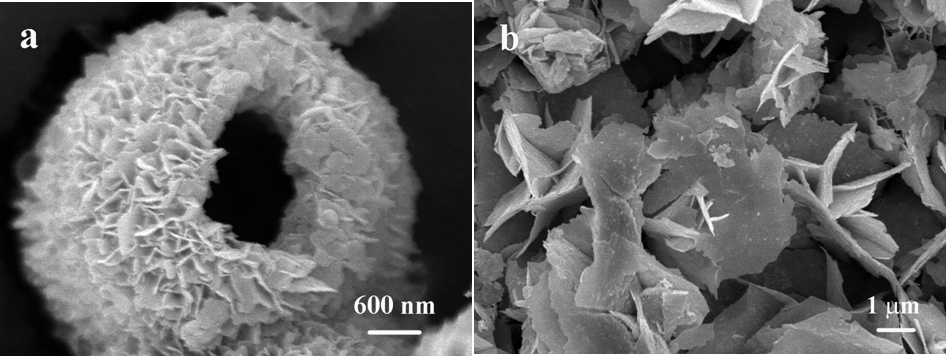


Figure S6 SEM images of the products prepared by treating BiOCOOH structures with tellurium sources under hydrothermal conditions


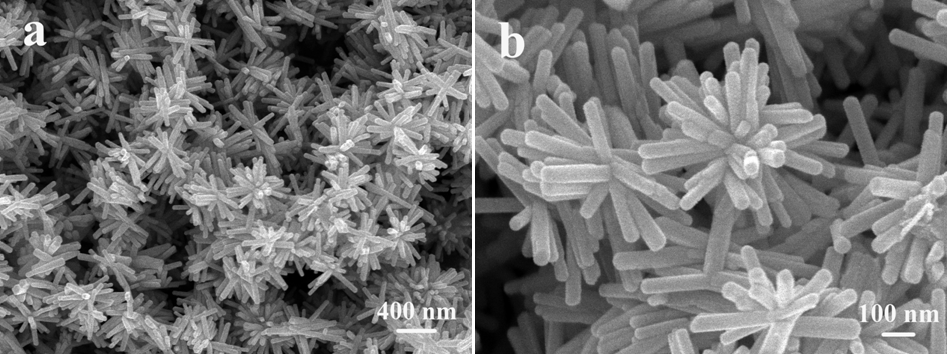


Figure S7 SEM images of comparative Bi_2_S_3_ nanostructures prepared by hydrothermal treating the aqueous solution of Bi(NO_3_)_3_ and thiourea at 160 °C for 16 h

Figures S8 Enlarged *M-H* curves from -2000 Oe ~ 2000 Oe of the obtained (a) hollow Bi_2_S_3_ spherical superstructures and (b) flower-like Bi_2_S_3_ superstructures
